# Summary of second annual MCBK public meeting: Mobilizing Computable Biomedical Knowledge—A movement to accelerate translation of knowledge into action

**DOI:** 10.1002/lrh2.10222

**Published:** 2020-03-01

**Authors:** Rachel L. Richesson, Bruce E. Bray, Christine Dymek, Robert A. Greenes, Leslie D. McIntosh, Blackford Middleton, Gerald Perry, Jodyn Platt, Christopher Shaffer

**Affiliations:** ^1^ Duke University School of Nursing Durham North Carolina; ^2^ University of Utah School of Medicine Salt Lake City Utah; ^3^ Agency for Healthcare Research and Quality Rockville Maryland; ^4^ Arizona State University and Mayo Clinic Scottsdale Arizona; ^5^ Research Data Alliance St. Louis Missouri; ^6^ Apervita, Inc. Chicago Illinois; ^7^ University of Arizona Health Sciences Library Tucson Arizona; ^8^ School of Medicine University of Michigan Ann Arbor Michigan; ^9^ University of California San Francisco San Francisco California

**Keywords:** clinical decision support, computable biomedical knowledge, dissemination, HIT policy, knowledge, metadata, standards

## Abstract

The volume of biomedical knowledge is growing exponentially and much of this knowledge is represented in computer executable formats, such as models, algorithms and programmatic code. There is a growing need to apply this knowledge to improve health in Learning Health Systems, health delivery organizations, and other settings. However, most organizations do not yet have the infrastructure required to consume and apply computable knowledge, and national policies and standards adoption are not sufficient to ensure that it is discoverable and used safely and fairly, nor is there widespread experience in the process of knowledge implementation as clinical decision support. The Mobilizing Computable Biomedical Knowledge (MCBK) community formed in 2016 to address these needs. This report summarizes the main outputs of the Second Annual MCBK public meeting, which was held at the National Institutes of Health on July 18‐19, 2019 and brought together over 150 participants from various domains to frame and address important dimensions for mobilizing CBK.

## 1 BACKGROUND

Despite significant healthcare spending in the United States, our health outcomes are worse than countries that spend far less.^1^ Morbidity and mortality data indicate growing health disparities,^2^ despite the (overwhelming amount of) accumulated knowledge from biomedical research. Undeniably, many factors cumulatively influence the health of our nation, but it appears that achieving the goal of routinely and equitably applying biomedical knowledge when and where it is needed remains elusive.

Biomedical knowledge is growing at a dramatic pace,^3^ and there is a pressing need to incorporate or provide access to this knowledge into a variety of systems, organizations, and applications. Increasingly, biomedical knowledge is represented in computable formats with potential for rapid dissemination.^4^, ^5^, ^6^ Computable biomedical knowledge (CBK), such as predictive models or executable rules, alerts, or data visualizations, can be integrated into health information systems to improve health care and the in‐context learning of trainees. CBK is essential for both the discovery and intervention components of learning health system activities.^5^ The movement to mobilize computable biomedical knowledge (MCBK) aims to represent knowledge in computable formats and make it widely available to achieve better health in different settings, contexts, and applications.

While there has been a great deal of research in knowledge sharing methods and techniques, only recently have more robust standards emerged for encoding and specifying knowledge in a computer interpretable form. This development, combined with the broad adoption of EHR technologies and the continuing pressures toward payment reform, have made the need for shareable computable knowledge paramount. The MCBK movement crystalized 3 years ago with a handful of clinical and informatics thought leaders who believed that the mobilization of CBK was essential to improving health and health care. In 2016, approximately 40 experts met at the University of Michigan to explore strategies to define requirements for infrastructure, standards, policies and best practices around CBK.^7^ In 2018, the First Annual MCBK public meeting was held at the National Library of Medicine and drew over 140 participants.^8^ Planners and participants developed a Manifesto (see *Supporting Information*) to articulate shared values and principles around CBK that would maximize the benefits, and minimize the harms, of widespread use of CBK for health, including imperatives to expose the underlying evidence and currency of CBK, and for efficient, safe, and equitable diffusion.^9^ Achieving the ideals of the Manifesto suggests an ecosystem with many actors and processes for generating new knowledge, evaluating it, applying it, and monitoring its effects. The MCBK movement is committed to shaping the development of an open ecosystem that will make CBK easily findable, accessible, interoperable, and reusable (FAIR).^10^

## 2 MEETING AND PARTICIPANT INFORMATION

The Second Annual MCBK public meeting was held July 18‐19, 2019 in the Natcher Conference Center at the National institutes of Health in Bethesda, MD. 195 people were registered and 162 attended, representing the following types of organizations: Universities/Academic Medical Centers (*n* = 71 [44%]), Government (*n* = 47 [29%]), Commercial/Industry and consultants (*n* = 26 [16%]), Professional societies (*n* = 9 [6%]), Health plans or providers (*n* = 6 [4%]), and Advocacy communities (for research, data or CDS; *n* = 3 [2%]).

### 2.1 Meeting structure and overview

A multidisciplinary Steering Committee^11^ guided the selection of topics and activities. The meeting included remarks from national leaders, panel presentations, poster sessions and technical demonstrations. The meeting included breakout sessions for work groups and an Open Mic session. The meeting agenda, list of speakers, registered participants and presentations are available at http://www.mobilizecbk.org.

### 2.2 Welcoming address and remarks from national leaders

Rachel Richesson and Charles (Chuck) Friedman opened the meeting with brief remarks on the evolution of the MCBK community and the MCBK Manifesto principles. They presented the meeting goals: to frame and address important dimensions for mobilizing CBK, advance work group action plans, and grow the MCBK community.

***Dr. Patricia Brennan***, Director of the National Library of Medicine (NLM), gave an opening keynote address and affirmed the importance of the CBK to the NLM's vision of a 21st century collection of biomedical knowledge to include literature, data, and models, as well as tools to make the aforementioned usable and useful for a wide range of users and stakeholders. Dr. Brennan emphasized NLM's commitment to making data sets FAIR^10^ and ready for advanced analytics. She spoke about models as the foundation of CBK and NLM's aspiration to advance science by supporting access to reproducible and re‐usable models. Dr. Brennan mentioned recent NIH guidance encouraging use of the Fast Healthcare Interoperability Resources (FHIR) standards specification for research applications and data sharing,^12^ and suggested that the unprecedented momentum around FHIR could energize the MCBK community and enable the use of CBK when and where it is needed.

***Dr. Don Rucker***, National Coordinator for Health Information Technology (ONC), shared his perspectives about defining standards and regulations to ensure that health data can be accessible for CBK and subsequent innovation. Dr. Rucker highlighted forces that will ultimately drive the mobilization of CBK, including exponential growth in computing power and data, new devices and consumer adoption, and an “economic revolution” in healthcare. He noted the prevalent national (and congressional) interest in consumer issues, such as drug pricing and surprise billing, and stated that transparent information around price—including analytic methods to compute and communicate price—will be essential to address these issues. Finally, Dr. Rucker noted that the ONC is working to facilitate the exchange of population level data (not just individual data exchange), which can be used by payers to quantify the value of healthcare activities and subsequently incentivize high‐quality care.

***Dr. Dipak Kalra***, President of The European Institute for Innovation through Health Data (*i*~HD),^13^ highlighted several initiatives in Europe as examples of how CBK can be used to improve research, learning health systems, and population health. Dr. Kalra suggested we learn from data standards communities' efforts to promote the adoption of complex standards. He described the *i*~HD's “Interoperability Asset Register”, which provides potential adopters with various *types* of resources, including *legal* (eg, policies and agreement templates), *organizational* (eg, adoption guidelines and training resources), *technical* (eg, information models and XML schema), and *semantic* (eg, clinical models and value sets).^14^ Dr. Kalra indicated that these types of resources will enable potential adopters to assess CBK artifacts and support implementation from both technical and organizational contexts. Finally, he challenged CBK developers to view themselves not simply as inventors, but as “founders” of knowledge communities that will continuously monitor and improve CBK.

### 2.3 Panel presentations

A ***Use Case Panel*** provided descriptive examples of how CBK can impact **public health**, **clinical**, **research**, and **education** domains. Dr. Shaun Grannis presented a brief overview of how CBK ‐ including machine learning and automatic case detection ‐ could be used to support automated public health reporting, a critical public health function that is labor intensive and often neglected. Dr. Marc Overage described multiple opportunities where CBK could improve the personalized management of pediatric asthma, including processing environmental data to predict exacerbations and suggesting preventive measures to both patients and providers. Dr. Jennie Larkin described the important role of CBK in the reproducibility and transparency of research, using as an example the dissemination and improvement of the award‐winning models arising from analytic “challenge” competitions designed to crowdsource solutions to important health problems. Dr. Anderson Spikard described a range of important applications for CBK in the area of health provider education, describing the use (and re‐use) of models that can customize and monitor learning activities for future generations of health professionals. (Slides and written summaries of the use cases are available at.^15^)

#### 2.3.1 Engaging critical stakeholders

A second panel session addressed critical stakeholder groups, including the **library community** and the biomedical knowledge **publishing industry**. Gerald (Jerry) Perry shared his perspectives about managing medical libraries and advocating for equitable access to information and participatory community research. He reminded the audience of the autonomy and self‐determination of communities impacted by disease and the importance of engaging them in the development and dissemination of knowledge. Mr. Perry suggested that the mobilization of CBK is not just about disseminating the knowledge, but also about enabling prudent use and accountability for that knowledge. He intimated the need for the MCBK community to expand its literacy around human behavior—ie, how people respond to new knowledge as it is consumed and utilized. He asserted that CBK should not just be FAIR,^10^ but also *fair* (ie, equitable) and transparent. This knowledge *fairness* must recognize that, while intended for good purposes, CBK can (intentionally or unintentionally) perpetuate discrimination and disparity, and these risks need to be considered as CBK algorithms and models become deployed and used in real‐world settings with diverse populations.

Nancy Allee, then Interim Director of the Taubman Health Sciences Library at University of Michigan, provided an overview of the current landscape for scholarly communication and described how the medical ***publishing industry*** is changing, leading to new business models and partnerships between academic medical libraries and publishers to support knowledge‐enabled health care. Ms. Allee highlighted the growing global support for the notion that research data should be accessible, re‐usable and citable (eg, the Research Data Alliance,^16^ World Data System,^17^ and CHORUS^18^), driven largely by desires to increase transparency and reproducibility of research. Additionally, she noted that software and models are being included with publications by a growing number of journals (including AI in Medicine, Computer Methods and Programs, PloS Computational Biology Software, and BiomedCentral, to name a few). Ms. Allee went on to describe a pilot program with the Wiley journal *Learning Health Systems (LHS)*,^19^ which (as of July 2019) began accepting submissions for CBK publications. The pilot program will use peer review to ensure trust, quality and reproducibility of the knowledge objects, and LHS editors will share their experiences (along with procedures, policies, and metadata) with the broader MCBK community.

### 2.4 Work group action sessions

The speakers described above provided background, vision, and motivation for meeting participants, who were charged to advance the MCBK vision through the four work groups formed during the first MCBK public meeting. Two breakout sessions (90 min each on days 1 and 2) were designated as Work Group Action Sessions. The work groups and their co‐chairs, scope, and discussions are summarized below.

The ***Standards Work Group***, led by Bob Greenes and Bruce Bray, is focusing on the identification of informatics standards specifications and metadata needed to support the application and FAIR knowledge capabilities for CBK. The work group is examining several CBK use cases that illustrate various types of CBK artifacts, and plans to identify metadata to characterize CBK and achieve FAIR goals. The metadata will include important dimensions (eg, source, purpose, intended user, domain) related to finding, accessing, interoperating and reusing CBK artifacts. One dimension of the metadata will likely include the data requirements of CBK objects, establishing linkages between standards for representing knowledge objects and clinical data representation standards. To this end, the group is exploring a map of relevant data standards that will expose areas where knowledge developers and implementers need guidance on how to combine various data standards and identify opportunities for harmonization and coordination of standards development efforts. This focus is on identifying and implementing existing standards rather than on developing new ones.

The ***Technical Infrastructure Work Group***, led by Leslie McIntosh and Chris Shaffer, is working to identify technical requirements for organizations to use and evaluate CBK. Their discussions at the meeting revolved around the primary question ‐ What are the framework components necessary to move CBK from generation into practice by facilitating the testing, versioning, use, evaluation, scalability, interoperability, and dissemination of CBK? A second question explored by the group was: how do we build and share a conceptual infrastructure model that supports mobilization and safe and effective use of CBK nation‐wide? Interoperability is a big issue and represents an area of synergy with the MCBK Standards Work Group. Moving forward, the Technical Infrastructure WG will identify very specific things that CBK developers will need to disseminate CBK across heterogeneous organizations and information systems. Group members are developing a Position paper focused on technical principles with real‐world examples and mini‐use cases.

The ***Policy and Coordination to Ensure Quality and Trust Work Group*** discussions were led by co‐chairs Jodyn Platt and Blackford Middleton. This work group builds upon the significant conceptual and consensus work of the Trust Framework Working Group in the area of clinical decision support.^20^ Their goal is to identify policy gaps and issues that impact the quality or trustworthiness of CBK. Topics discussed during breakout sessions included how to describe or catalog the landscape of CBK and what policy and coordination will be needed to support a “knowledge commons ecosystem”^21^ that engages and balances the interests of public and private entities with accessibility and sustainability of usable CBK. A knowledge commons ecosystem carries inherent requirements for governance and trust to manage risk and facilitate the use of CBK by end users in clinical and community environments. Currently, the work group is completing and reporting results of landscape analysis, identifying market considerations, including: (a) different representations (narrative to semi‐structured to executable^22^) for knowledge artifacts, (b) current business models for knowledge creation and dissemination, (c) governance strategies, and (d) organizational culture for trust and use of this knowledge in real settings. Several members of the MCBK Policy and Coordination Work Group collaborate with the AHRQ evidence‐based Care Transformation Support (ACTS) initiative.^23^

The ***Sustainability for Mobilization and Inclusion Work Group***, chaired by Christine Dymek and Jerry Perry, is curating a list of important MCBK stakeholder groups, and developing strategies and approaches to message and engage with each. Important stakeholders include knowledge creators, knowledge curators, knowledge distributors, and knowledge engineers, as well as technology developers, people in professional societies and generators of clinical guidelines. Dr. Dymek and Mr. Perry are committed to identifying and including other critical stakeholders, including patient advocacy groups and patients, who represent ultimate end users of CBK, and policy makers, many of whom are aware of the need for oversight or good practices for managing CBK (eg, the proposed “Algorithm Accountability Act”.^24^) As a first activity, the Sustainability work group is using information from four interviews to develop targeted messages and communication strategies for affinity professional societies.

### 2.5 Posters and technical demonstrations

There were 24 posters and 17 technical demonstrations—nearly double that of the first meeting—and many represented mature or active CBK use and tools. Of the 17 technical demos, 6 (35%) came from commercial entities, 9 (53%) from academia, and 2 (12%) from standards development organizations. Collectively, they represented the perspectives of knowledge developers, disseminators, and users. A good number of technical demonstrations represented actual implementations of CBK. Of the 24 posters, 18 (75%) came from academia, 4 (17%) from government, 1 (4%) from a commercial entity and 1 (4%) from a standards development organization. Broadly speaking, the posters also represented the perspectives of knowledge developers, disseminators, and users, and can be viewed here: https://medicine.umich.edu/sites/default/files/content/downloads/Poster%20Combined_0.pdf

### 2.6 Reflections panel

The meeting was closed with a Reflections Panel from invited members of the MCBK Steering Committee. Dr. Leslie McIntosh summarized the speakers and discussions over the 2‐day meeting, which provided a foundation for us to reflect on our evolving discussions. Dr. McIntosh challenged us to think about who was present (and who wasn't) and encouraged us to actively watch other groups that are doing complementary work. Dr. Peter Embi shared his insight and perspectives from his recent experience *as a patient*, noting that his good outcome was largely the result of his knowledge and position as a medical doctor, but that CBK could have improved the speed of diagnosis for him and others in his situation. Dr. Embi also asserted the need to monitor the safety and impact of CBK knowledge after it is deployed, calling for *“Algorithmovigilance”* (analogous to pharmacovigilance) methods and programs to identify and address unintended consequences of CBK.

Doug Van Houweling shared his thoughts (gleaned from decades of experience watching new movements, organizations, and disruption), which served as a ruminative conclusion to the meeting and this report. He commented on the positive change in tone and productivity from the first meeting, illustrated by the increase in technical demonstrations from last year and the number of first‐time, MCBK participants bringing diverse ideas and rich discussion to the meeting. Dr. Van Houweling challenged the audience to contemplate how to harness this collective expertise moving forward, and to clarify the role and function of the MCBK community. The convening power of the MCBK community and importance of mobilizing CBK was evidenced by the large meeting attendance, but how should MCBK evolve? Should MCBK serve to coordinate important CBK stakeholders and relevant activities? Define and endorse standards for CBK? Support demonstrations and applications of CBK? Curate CBK? Provide infrastructure? Promote policy? Dr. Van Houweling stated that any one of these functions could be useful, but “the community” needs to decide. Whatever that choice, the next step for MCBK will be to define value propositions for various stakeholders, and develop plans to communicate, engage, and work with potential partners and sponsors. Although the MCBK community is just 2 years old, we will inevitably need to consider governance, funding, resources, and future utility and sustainability of MCBK in the bigger world.

### 2.7 Findings and Impressions (from the authors)

The number and diversity of meeting attendees indicates that MCBK fills an important but broad niche. As planners and participants in the meeting, we witnessed autonomy, vision, and momentum in the MCBK community. Work group chairs and members are enthusiastic to continue their discussions and activities into the next year. There was general support for subsequent public meetings.

Many meeting attendees were new to the MCBK vision and community, and as a result our work groups spent significant time discussing the scope of the work and different perspectives, ideas, and interests of those participating. This is a known and inherent part of the collaborative process. Coalescing multidisciplinary stakeholders toward a common language and goals in a dynamic and complex ecosystem will be an ongoing challenge, but will be worthwhile if we realize a knowledge ecosystem where CBK is applied routinely, safely, and equitably to improve our health and healthcare experience.

### 2.8 Next steps and FUTURE for MCBK

The University of Michigan will continue to provide communications support for MCBK workgroups and their members. Two webinars were presented in Fall 2019 to summarize the meeting and use cases. Plans for a Third Annual MCBK public meeting for Summer 2020 are underway. The MCBK is an open and inclusive community. Anyone that is interested in joining an MCBK work group may sign up here: http://mobilizecbk.org/.

## CONFLICT OF INTEREST

Blackford Middleton is employed by Apervita, Inc., which provides a platform and marketplace to democratize healthcare data and analytics. Leslie McIntosh is founder and CEO of Ripeta, LLC, a company providing reproducibility checks on scientific manuscripts. Gerald (Jerry) Perry served on the Board of Directors for the Association of Academic Health Sciences Libraries (AAHSL) during the period of work described in this article. AAHSL is one of the four professional societies of whose members were interviewed to develop targeted communications strategies for affinity professional societies, as a component of the Sustainability for Mobilization and Inclusion Work Group's efforts. Mr. Perry has no competing commercial interests. The other authors (Rachel Richesson, Bruce Bray, Christine Dymek, Robert Greenes, Leslie McIntosh, Jodyn Platt, and Christopher Shaffer) have no competing interests to declare.

## AVAILABILITY OF DATA AND MATERIALS

Videos and slides from the plenary sessions, use cases, and the posters and technical demonstrations presented, are posted on the website: https://medicine.umich.edu/dept/lhs/2019-mcbk-meeting.

## Supporting information


**Appendix** S1: Supporting Information
