# Supplementary material for: Summary of second annual MCBK public meeting: Mobilizing Computable Biomedical Knowledge—A movement to accelerate translation of knowledge into action
Source: Learn Health Syst. 2020 Mar 1;4(2):e10222. doi: 10.1002/lrh2.10222 (PMC7156866; doi:10.1002/lrh2.10222)
Supplement: Supplementary file 1 — Appendix S1: Supporting Information [file LRH2-4-e10222-s001.pdf]

Mobilizing Computable Biomedical Knowledge (CBK): A Manifesto

Preamble

Knowledge has the potential to improve health care, the health of individuals, and the health of populations. Every decision affecting health should be informed by the best available knowledge. For moral and ethical reasons, it is imperative that each and every member of society has access to what is known at the time they are making health-related choices and decisions.

It is no longer sufficient to represent knowledge in the form of printed words and static pictures. The increasingly rapid rate of scientific discovery needs knowledge representations that are more agile and amenable to scalability and mass action. This in turn can enable the continuous cycles of discovery and improvement envisioned as Learning Health Systems.

Contemporary digital technology enables knowledge to be represented in *computable* forms expressed in machine-executable code. Computable knowledge unleashes the potential of information technology to generate and deliver useful information—and particularly, decision-specific advice--to individuals and organizations with great speed on a world-wide scale. It is essential to take full advantage of these capabilities, while continuing established practices that validate knowledge, preserve it, and ensure that it can be trusted.

There is work to do to mobilize best available health knowledge for the greater good. To begin, biomedical knowledge in computable form must be made interoperable using open standards, and widely available so that it can be used to immediately impact health.

It is time for action on a global scale.

Computable Biomedical Knowledge

Computable Biomedical Knowledge is the result of an analytic and/or deliberative process about human health, or affecting human health, that is explicit, and therefore can be represented and reasoned upon using logic, formal standards, and mathematical approaches.

Vision

We are dedicated to:

Mobilizing biomedical knowledge that can support action toward improving human health. This should be done using computable formats that can be shared and integrated into health information systems and applications.

Efficiently and equitably serving the learning and knowledge needs of all participants, as well as the public good. This will work to significantly reduce health disparities.

Ensuring that the knowledge properly reflects the best and most current evidence and science. This will ensure that knowledge can be trusted for use to improve health and health care.

*Achieving this through evolution of an open Computable Biomedical Knowledge ecosystem dedicated to achieving the FAIR principles: making Computable Biomedical Knowledge easily findable, universally accessible, highly interoperable, and readily reusable.** The current interest in making data "FAIR" should be matched by equally intense interest in making knowledge "FAIR".

Mechanisms of Activity

We believe that all of the following are important:

- The CBK Concept
    - Sustain the Computable Biomedical Knowledge ecosystem through public-private partnerships.
    - Establish broadly-based participatory governance of the ecosystem.
    - Make the ecosystem diverse and inclusive.
    - Explore the sciences of Computable Biomedical Knowledge collaboratively.
    - Be agile to reflect the increasingly rapid changes in knowledge.
- The CBK Technical System
    - Enable the ecosystem with open standards.
    - Build and uphold trust in Computable Biomedical Knowledge through the ecosystem.
    - Ensure robust and unbiased methods to support transparency and expose the currency, validity and provenance of Computable Biomedical Knowledge.
    - Implement the highest standards of privacy and security for all stakeholders.
    - Enable a pipeline that transitions knowledge from human-readable to fully computable through successive stages.
- The CBK Use/User System
    - Ensure the safe and effective use of Computable Biomedical Knowledge through the ecosystem.
    - Generate value for the creators of the knowledge, the users of the knowledge, and the general public.
    - Engender equity in health and in knowledge accessibility

*- Wilkinson MD, Dumontier M, Aalbersberg IJ, Appleton G, Axton M, Baak A, Blomberg N, Boiten JW, da Silva Santos LB, Bourne PE, Bouwman J. The FAIR Guiding Principles for scientific data management and stewardship. Scientific data. 2016;3.
